# Hidden burden of leprosy in incarcerated populations in northeast Brazil: active case detection and serological assessment

**DOI:** 10.1007/s10096-026-05578-2

**Published:** 2026-07-13

**Authors:** Thatiane Silva de Lucena, Lorena Covre Galani, Luize Dianin Barbosa, Gabriela Ferreira Dias, Jaci Maria Santana, Filipe Rocha Lima, Marco Andrey Cipriani Frade

**Affiliations:** 1 Division of Dermatology, Department of Internal Medicine, Ribeirão Preto Medical School, University of São Paulo, São Paulo, Brazil; 2 National Referral Center for Sanitary Dermatology focusing on Leprosy, Clinical Hospital of the Ribeirão Preto Medical School, University of São Paulo, São Paulo, Brazil; 3 Laboratory for Skin Studies and Alternative Models, Department of Internal Medicine, Ribeirão Preto Medical School, University of São Paulo, São Paulo, Brazil; 4 Baldomero Cavalcante de Oliveira Penitentiary, Department of Penitentiary Administration, Maceió, Alagoas Brazil; 5 Department of Biochemistry and Immunology, Ribeirão Preto Medical School, University of São Paulo, Ribeirão Preto, São Paulo Brazil; 6 Leprosy Laboratory, Otávio de Freitas Hospital, Pernambuco State Health Secretariat, Recife, Pernambuco Brazil; 7 Black Skin Outpatient Clinic, Otávio de Freitas Hospital, Pernambuco State Health Secretary, Pernambuco, Brazil

**Keywords:** Leprosy, Hansen’s disease, Prison population, Active case finding, Serological screening

## Abstract

**Background:**

Leprosy remains a public health challenge in Brazil’s Northeast, where prison overcrowding may facilitate transmission. Limited data exist on leprosy screening strategies in incarcerated populations.

**Methods:**

This observational, cross-sectional study was conducted at Baldomero Cavalcante Male Penitentiary in Maceió, Alagoas, Brazil. A total of 915 inmates were approached using the Leprosy Suspicion Questionnaire (LSQ) as an active case-finding tool. Neurodermatological examinations were performed on 260 participants, and serological testing for anti-PGL-I (IgM) and anti-Mce1A antibodies (IgA, IgM, IgG) was conducted on 244 plasma samples using indirect ELISA.

**Results:**

Of 842/915 returned questionnaires (92%), 240/842 (28.5%) were positive in the LSQ. Clinical examination revealed 8 new leprosy cases among 260/842 evaluated inmates with positive and negative LSQ, yielding an overall new case detection rate (NCDR) of 3.08%, which increased to 6.52% among symptomatic individuals. All diagnosed patients were classified as having multibacillary disease, with 100% presenting some grade of physical disability at diagnosis, and 75% LSQ were positive (*p* = 0.0249), while only 34.1% in the non-leprosy group. Anti-Mce1A IgM showed 37.7% seropositivity rate, and 19.9% for anti-PGL-I among clinically unaffected individuals. IgA (100%), IgM (37.5%), and IgG (25%) anti-Mce1A antibodies were positive in the leprosy group.

**Conclusions:**

The LSQ may be effective as a screening tool. The study confirms significant underdiagnosis of leprosy in prison populations and highlights that anti-Mce1A serology showed higher positivity among diagnosed cases than anti-PGL-I, particularly for the detection of early-stage and neural forms of the disease. These findings support enhanced active surveillance strategies in high-risk populations to achieve leprosy elimination goals.

**Supplementary Information:**

The online version contains supplementary material available at https://doi.org/10.1007/s10096-026-05578-2.

## Introduction

Leprosy is an infectious disease caused by *Mycobacterium leprae* and *Mycobacterium lepromatosis*, which primarily affects peripheral nerves and the skin, as well as ocular, nasal, articular, lymphatic, visceral, and bone marrow tissues, leading to neuropathy and other consequences, including disabilities and deformities [1–4]. According to World Health Organization (WHO) data, in 2023, a total of 182,815 new cases of leprosy were reported worldwide, of which 22,773 occurred in Brazil, accounting for 92% of all cases in the Americas and placing the country second in absolute number of cases [5]. In this context, the Brazilian Northeast is one of the regions with the highest number of leprosy cases, with a total of 146,014 reported between 2012 and 2021 [6]. According to the 2025 epidemiological report, the Central-West, North, and Northeast regions recorded the highest detection rates of the disease [7].

Recent studies have demonstrated the existence of hidden leprosy endemicity in Brazil, including in regions officially considered non-endemic. This situation is largely related to the limited diagnostic capacity of health professionals, as leprosy diagnosis still relies mainly on the identification of clinical signs and symptoms [8]. For decades, public health policies regarding diagnosis have prioritized the search for dermatological alterations to the detriment of neurological symptoms associated with infection [8, 9]. It is understood that implementing active case-finding strategies grounded in theoretical and practical training, the application of the LSQ, and the provision of diagnostic support can lead to a 300% increase in the annual number of reported cases in areas classified as having low endemicity [10]. This underscores the importance of active surveillance and additional case detection among household contacts of newly diagnosed cases by experienced dermatologists [11].

Since 2000, the prison population has increased substantially, with South America experiencing a 60,5% rise [12], where Brazil holds the third largest number of incarcerated individuals worldwide, and in 2025, 94.13% of the inmates are male [13, 14]. According to the National Survey of Prison Information (Infopen) 2025, the Brazilian prison system operates with a deficit 30.5%, housing 727,301 inmates for only 505,267 available places [13]. This situation has worsened over time compared to previous reports, such as Infopen 2021, demonstrating the persistence of structural deficiencies in the prison system [15], mainly due to factors related to the prison environment, among other socioeconomic factors [16]. In 2025, the prison population in Brazil’s Northeast region accounts for 17.53% of the national incarcerated population, with 20.1% (44,693/222,034) of inmates exceeding the national system’s capacity [13]. This scenario highlights the widespread problem of prison overcrowding worldwide [17]. Furthermore, overcrowding increases susceptibility to the transmission of infectious diseases such as leprosy [14, 18, 19]. This highlights the importance of integrated control and active epidemiological surveillance for the effective control of leprosy, with new diagnostic and treatment approaches [20, 21].

Although there are numerous studies about tuberculosis (TB) in the prison system with well-established risks [22], there are still few studies about leprosy in the prison and socioeconomically disadvantaged population [23–25].

Therefore, this study was conducted to evaluate effective tools for screening new leprosy cases, including the LSQ and serological biomarkers associated with exposure and contact with the bacillus, and the development of active disease. In addition, it seeks to characterize clinical, immunoepidemiological, and social factors associated with the disease, and to determine the true detection rate of new cases in the prison environment, with the objective of interrupting the transmission chain in this population.

## Methods

### Location and population of the study

This is an observational, cross-sectional, and comparative study conducted in a prison complex located in Maceió, the capital city of the state of Alagoas in northeastern Brazil, which has a population of 994,952 inhabitants and reported a leprosy detection rate of 11.27 cases per 100,000 inhabitants in 2023. The state of Alagoas was classified as a high endemic region for leprosy in 2023. Within this complex, the Baldomero Cavalcante Male Penitentiary unit was selected, which, according to the latest data from June 2020, had a capacity for 773 inmates but housed 915 inmates and is considered a high-security facility [26, 27].

### Strategy for active case finding

The prisoners were administered the LSQ, consisting of 14 objective questions addressing key signs and symptoms of the disease, which was developed by the Brazilian leprosy reference center (CRNDSHansen HCFMRP-USP) and described by Bernardes Filho et al. (2020) [14].

Participants were informed only that the questionnaire addressed signs and symptoms and that they should mark the items according to what they experienced, without receiving any explanation of the disease to avoid bias in their responses. Illiterate individuals received assistance with reading and writing from the nursing technician and the physician responsible for patient care. The questionnaire was distributed throughout the prison population. Before clinical evaluation, inmates who agreed to participate signed informed consent forms, returned the questionnaires, and subsequently underwent a clinical-dermatological examination. In addition, questionnaires on habits and customs, with an emphasis on social and environmental aspects, were administered during the assessment.

The prison is organized into sectors, each consisting of corridors (referred to as wings), each containing different cells. Inmates from each wing and their respective cells were escorted by prison officers to the sector’s common area, where the research team conducted the study procedures. The researchers were previously informed of the prison sectors in which inmates had completed the LSQ questionnaire, regardless of their LSQ status. Both LSQ-positive and LSQ-negative inmates who agreed to participate in the study were included. After blood sample collection, all participants underwent clinical dermato-neurological evaluation conducted by dermatologists and leprologists, with nursing support. A total of 260/842 inmates were evaluated during the study period. The limited number of participants was due to security restrictions and guidance from the prison administration following the identification of a potential riot situation.

### Diagnostic criteria for leprosy

Active case-finding for leprosy was conducted in accordance with the guidelines established in the Brazilian Municipal Manual of Care for People with Leprosy [28]. Thermal, pain, and tactile sensitivity tests were performed, along with esthesiometry of the hands and feet using Semmes-Weinstein monofilaments (SWM) [29] and sensory mapping of skin lesions [30], when present. The diagnosis of leprosy was established based on the cardinal signs defined by the WHO, with at least one of the following: (i) definite loss of sensation in a hypopigmented or erythematous skin patch; (ii) thickened or enlarged peripheral nerve with associated sensory loss and/or muscle weakness in the area supplied by that nerve; or (iii) the presence of acid-fast bacilli in slit-skin smears [2].

Given that no single classification system fully encompasses the entire spectrum of clinical manifestations of leprosy, particularly the macular and pure neural forms, patients were classified based on adapted criteria from the Ridley-Jopling [31], Madrid [32], and Indian Association of Leprology [33] schemes as follows: indeterminate (I), polar tuberculoid (TT), borderline tuberculoid (BT), borderline borderline (BB), borderline lepromatous (BL), polar lepromatous (LL), and pure neural (N). Additionally, according to operational criteria [28]: paucibacillary (PB, corresponding to I, TT, BT) and multibacillary (MB, corresponding to BB, BL, LL, and N). According to the WHO, cases were classified as MB based on the following indication: a case of leprosy with more than five skin lesions or with nerve involvement (pure neuritis or any number of skin lesions and neuritis) [34, 35]. All newly diagnosed patients were referred to a health unit for standard multidrug therapy (MDT).

### Assessment of anti-PGL-I and anti-Mce1A titer by ELISA

The IgA, IgM, IgG anti-Mammalian Cell Entry Protein 1 A (Mce1A) and IgM anti-Phenolic Glycolipid-I (PGL-I) antibodies of 244 plasma samples from patients were measured by indirect enzyme-linked immunosorbent assay (ELISA). The recombinant Mce1A protein and PGL-I [natural-octyl disaccharide conjugated to bovine serum albumin (ND-O-BSA; NR-19346; BEI Resources)] assays were applied as described by Lima et al. (2023) [36]. The index was calculated by dividing the mean O.D. by the established cut-off, and values greater than 1.0 were considered positive, with risk groups stratified according to the patient’s positivity index: values between 1.0 and 1.5 were classified as low risk, between 1.5 and 2.0 as moderate risk, and values above 2.0 were considered high risk for leprosy positivity.

### Statistics analysis

Statistical analysis of the data was performed using GraphPad Prism version 10.0 (GraphPad Inc., La Jolla, CA, United States). Normality was assessed using the D’Agostino & Pearson, Shapiro–Wilk, and Kolmogorov–Smirnov tests, and the data were classified as nonparametric. Variations in antibody levels were analyzed using the Kruskal–Wallis test followed by Dunn’s post hoc test, while differences between groups were evaluated using the Mann–Whitney U test, along with their respective 95% confidence intervals (CI), Z scores, and effect sizes (r or *n*^2^_H_). Categorical variables were analyzed using Fisher’s exact test, and their respective relative risk (RR) with CI. The level of statistical significance was set at α = 0.05, with *p* < 0.05 considered significant. The data evaluated are available in Supplementary Material 1 (raw data and database).

## Results

### Screening of individuals and case detection

**Fig. 1:**
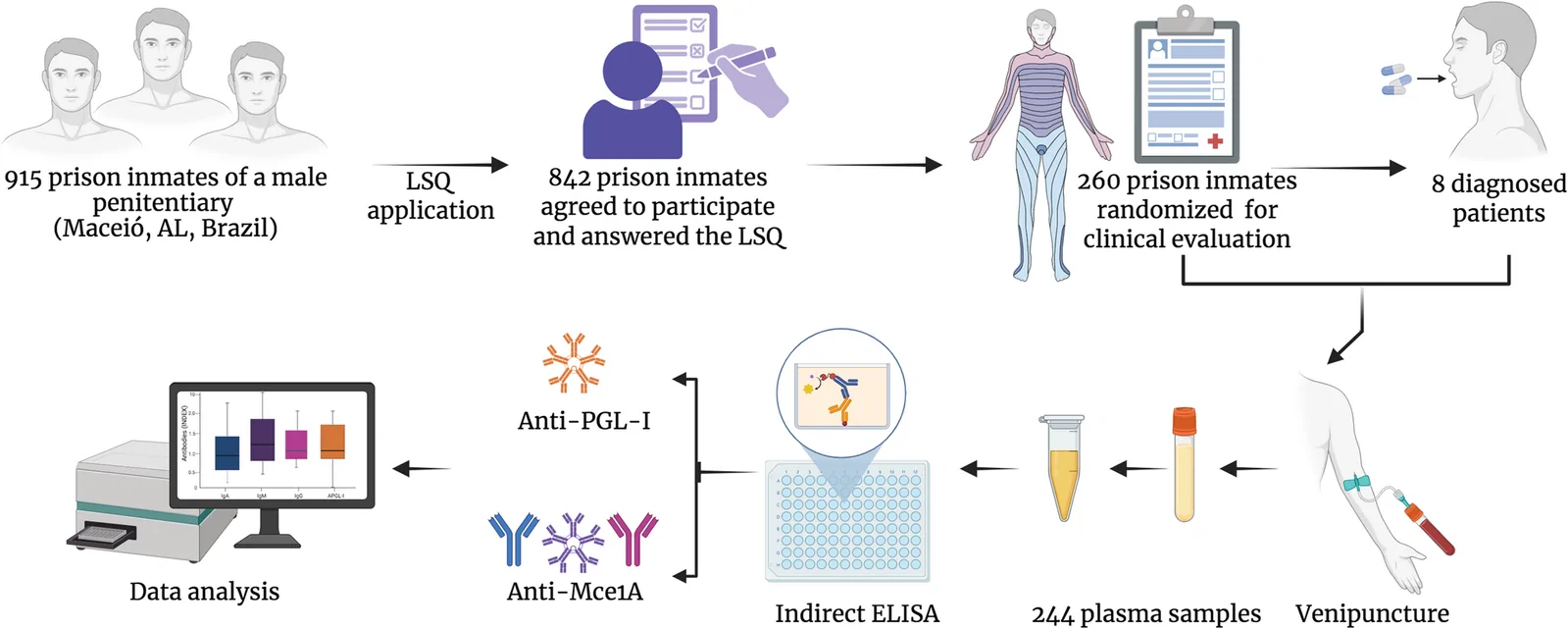
Research strategy of active search for new cases. Flowchart of questionnaire application, clinical evaluation of individuals, processing of blood samples, execution of serological tests, and data analysis. Created in BioRender. Lima, F. (2026)

A total of 915 questionnaires were distributed to the study population, of which 842 were returned, corresponding to 92% of the total. Among the returned questionnaires, 240/842 were positive for signs or symptoms of leprosy, representing 28.5% of the total, with an average of 3.05 positive responses per person. The research strategy is outlined in Fig. 1.

A clinical examination was conducted on 260/842 (30.9%) inmates, LSQ-positives and LSQ-negatives, of whom 8/260 were diagnosed with leprosy by at least two dermatologists, resulting in an NCDR of 3.08% in this population. Patients diagnosed with leprosy were assigned to the L group (*n* = 8/260), and those without a leprosy diagnosis were assigned to the NL group (*n* = 252/260). Among those evaluated, 92 had a positive questionnaire for signs or symptoms of leprosy, and 6 of them were clinically diagnosed, yielding an NCDR of 6.52%. This represents a doubling of the NCDR when considering only cases with positive screening questionnaires, thereby increasing the efficiency of the intervention. L group had an average of 2.6 positive responses on the questionnaire, with “numbness in hands and feet” [question 1; *p* = 0.15; RR = 2.14 (CI = 0.76–4.32)] and “nerve pain” [question 7; *p* = 0.09; RR = 2.33 (CI = 0.92–4.70)] being the most frequent symptoms. [75% (6/8) of patients in the L group had positive LSQ results], and 34.1% (86/252) in the NL group [*p* = 0.0249; RR = 2.19 (CI = 1.17–2.97)]. Facial swelling was observed in 33.3% (2/8) of patients in the L group [*p* = 0.0079; RR = 21.0 (CI = 4.35–88.25)] (Table 1).

**Table 1:** Distribution of inmates clinically evaluated according to the questions marked on the LSQ in the penitentiary

LSQ responses, *n* (%)	L group *n* = 8	NL group *n* = 252	Total *N* = 260	*p* value^a^
Number of positives LSQ, n (%)	6 (75.0)	86 (34.1)	92 (35.4)	**0.0249**
1. Do you feel numbness in your hands or feet?	3 (50)	44 (51.2)	47 (51.1)	0.1594
2. Tingling?	1 (16.7)	55 (63.9)	56 (60.9)	> 0.9999
3. Anesthetized areas on the skin?	2 (33.3)	21 (24.4)	23 (25.0)	0.1511
4. Spots on the skin?	1 (16.7)	29 (33.7)	30 (32.6)	> 0.9999
5. Stinging sensation?	1 (16.7)	16 (18.6)	17 (18.5)	0.4223
6. Skin nodules?	0 (0)	13 (15.1)	13 (14.1)	> 0.9999
7. Nerve pain?	4 (66.7)	36 (41.9)	40 (43.5)	0.0992
8. Swelling of hands or feet?	1 (16.7)	9 (10.5)	10 (10.9)	0.2725
9. Swelling of the face?	2 (33.3)	3 (3.5)	5 (5.4)	**0.0079**
10. Weakness in the hands?	2 (33.3)	26 (30.2)	28 (30.4)	0.2086
11. Trouble buttoning your shirt? Putting on your glasses? Writing? Holding pots and pans?	2 (33.3)	12 (13.9)	14 (15.2)	0.0627
12. Weak feet? Difficulty putting on sandals/flip-flops?	2 (33.3)	17 (19.8)	19 (20.6)	0.1088
13. Loss of eyelashes?	0 (0)	8 (9.3)	8 (8.7)	> 0.9999
14. Loss of eyebrows?	0 (0)	4 (4.6)	4 (4.3)	> 0.9999

Statistical comparison between group leprosy (L) and group non-leprosy (NL). ^a^Fisher’s exact test

### Sociodemographic characterization

L group predominantly comprised individuals aged 30 to 59 years (37.5%) and over 60 years (37.5%). 75% of patients had educational attainment classified as illiterate or incomplete primary education, 100% were born in the Brazilian state of Alagoas, 75% were single, 50% had one Bacillus Calmette-Guérin (BCG) scar, and the median duration of incarceration was 40 months. In contrast, NL group had a higher proportion (48%) of individuals aged 30 to 59 years, with the majority also classified as illiterate or having incomplete primary education; 89.7% were born in Alagoas, 48.8% were single, 66.3% had one BCG vaccination, and the median duration of incarceration was also 40 months, although the maximum duration reached up to 264 months (Table 2). No statistically significant differences were observed between the groups evaluated.

**Table 2:** Sociodemographic characteristics of inmates clinically evaluated in the penitentiary

	L group *n* = 8	NL group *n* = 252	Total *N* = 260	*p* value
Age range, years, n (%)				0.0974ª
< 30	2 (25.0)	116 (46.0)	118 (45.4)	
30–59	3 (37.5)	121 (48.0)	124 (47.7)	
≥ 60	3 (37.5)	15 (6.0)	18 (6.9)	
Education, n (%)				
Illiterate and incomplete elementary school	6 (75.0)	172 (68.3)	178 (68.5)	> 0.9999^b^
Complete elementary and incomplete high school	2 (25.0)	57 (22.6)	59 (22.7)	> 0.9999^b^
Complete secondary education and incomplete higher education	0 (0)	22 (8.7)	22 (8.5)	> 0.9999^b^
Higher education	0 (0)	1 (0.4)	1 (0.4)	> 0.9999^b^
Brazilian state of birth, n (%)^c^				
Alagoas	8 (100)	226 (89.7)	234 (90.0)	> 0.9999^b^
São Paulo	0 (0)	9 (3.6)	9 (3.5)	> 0.9999^b^
Pernambuco	0 (0)	11(4.4)	11 (4.2)	> 0.9999^b^
Sergipe	0 (0)	5 (2.0)	5 (1.9)	> 0.9999^b^
Paraíba	0 (0)	1 (0.4)	1 (0.4)	> 0.9999^b^
Marital status, n (%)				
Single	6 (75.0)	123 (48.8)	126 (48.5)	0.1704^b^
Married/living together	2 (25.0)	109 (43.2)	111 (42.7)	0.4727^b^
Common-law relationship	0 (0)	8 (3.2)	8 (3.1)	> 0.9999^b^
Divorced	0 (0)	8 (3.2)	8 (3.1)	> 0.9999^b^
Widowed	0 (0)	2 (0.8)	2 (0.8)	> 0.9999^b^
BCG scar, n (%)				
0	3 (37.5)	54 (21.4)	57 (21.9)	0.3786^b^
1	4 (50.0)	167 (66.3)	171 (65.8)	0.4513^b^
2	1 (12.5)	31 (12.3)	32 (12.3)	> 0.9999^b^
Time of deprivation of liberty				0.9559ª
Months, median (min-max)	40 (15–108)	40 (1-264)^d^	40 (1-264)	

Statistical comparison between group leprosy (L) and group non-leprosy (NL). ªMann-Whitney test. ᵇFisher’s exact test. ^c^Data not evaluated for two individuals in the NL group. ^d^Data not evaluated for three individuals. BCG: Bacillus Calmette-Guerin. *SD,* standard deviation. *Min,* minimum. *Max,* maximum

### Clinical characterization of detected cases

**Fig. 2:**
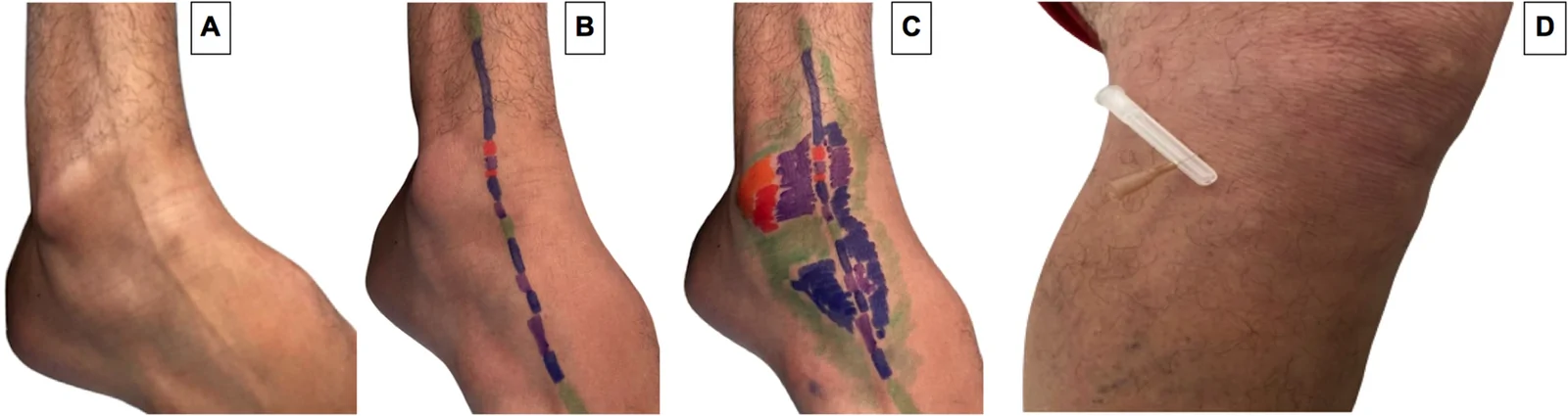
Images of the neurodermatological examination. **A** Thickening of the superficial fibular nerve of the left foot. **B** Altered tactile sensitivity testing along the course of the superficial fibular nerve by SWM. **C** Sensory mapping in the region of the left superficial fibular nerve showing an island of altered sensitivity to blue (0.2 g-force), violet (2 g-force), red (4 g-force), orange (10 g-force) by SWM, delimited by a green area of normal sensitivity (0.07 g-force). **D** Poorly delimited hypochromic macula with rarefied hair and loss of thermal, pain, and tactile sensitivity in the right leg. Image showing pain sensitivity testing with a 13 × 0.45 mm needle

The L group was classified as MB (100%); 75% were classified as B-form and 25% as N-form. Approximately 62.5% exhibited dysesthesia without any classical cutaneous lesions, and 100% showed neural involvement. All patients exhibited some grade of physical disability (GPD) at the time of diagnosis, with 87.5% classified as Grade 1 disability (G1D) and 12.5% as Grade 2 disability (G2D) (Table 3). The presence of hypopigmented and hypoesthetic macules, and sensitive mapping along the superficial fibular nerve and the overlying skin, were illustrated using SWM (Fig. 2).

**Table 3:** Clinical characteristics of inmates evaluated at the Penitentiary (*n* = 8)

Clinical criteria, *n* (%)	
Dysesthesia with skin lesion	3 (37.5)
Dysesthesia without skin lesion	5 (62.5)
Hair thinning	3 (37.5)
Affected nerves	8 (100.0)
Grade of Physical Disability, n (%)	
0	0 (0)
1	7 (87.5)
2	1 (12.5)
Clinical Form, n (%)	
B	6 (75.0)
N	2 (25.0)
Operational Classification, n (%)	
PB	0 (0)
MB	8 (100)

*B,* borderline; *N,* pure neural; *PB,* paucibacillary; *MB,* multibacillary

### Serological assay and biomarker performance

**Fig. 3:**
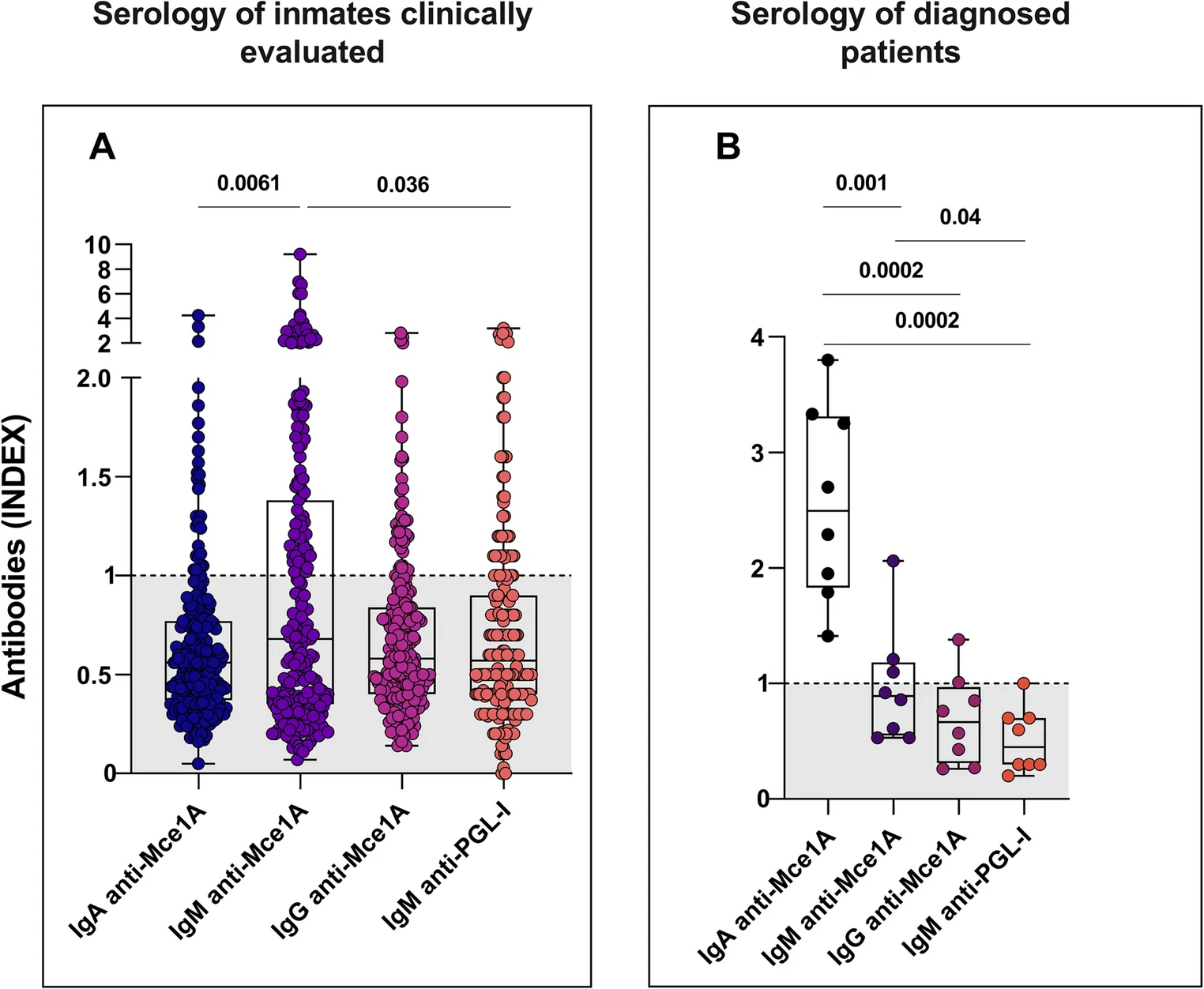
Serological Biomarkers for leprosy screening. **A** IgA, IgM and IgG antibodies indices against Mce1A protein and IgM anti-PGL-I in samples from non-leprosy group. Schematic representation of community transmission highlighting case detection. **B** antibodies indices in samples from leprosy group. Statistical significance was determined by the Kruskal–Wallis test followed by the Dunn’s test correction (multiple groups) and Mann-Whitney U test (two groups). Data are presented as the median and interquartile range (IQR). The respective index was calculated by dividing the optical density (O.D 450 nm) of each sample by the cut-off, and indices above 1.0 were considered positive represented by horizontal dotted line

Anti-Mce1A and anti-PGL-I (APGL-I) ELISAs were performed on plasma samples from *N* = 244 clinically evaluated inmates, including *n* = 236 NL group and *n* = 8 L group. Among individuals of the NL group, anti-Mce1A IgA was positive in 12.3% [29/236; median = 0.56; interquartile range (IQR) = 0.37–0.77; CI = 0.59–0.70], with 1.3% (3/236) exhibiting indices > 2.0. The IgM antibody showed the highest seropositivity at 37.7% (89/236; median = 0.68; IQR = 0.35–1.38; CI = 0.91–1.22), and 12.7% (30/236) had indices > 2.0, which was statistically significant compared to the IgA ELISA (*P* = 0.0061; Z = 2.94) and APGL-I (*P* = 0.03; Z = 2.00). The IgG ELISA was positive in 15.7% of tested samples (37/236; median = 0.58; IQR = 0.40–0.84; CI = 0.63–0.73), and1.7% (4/236) with indices > 2.0. APGL-I serology showed 19.9% seropositivity (47/236; median = 0.57; IQR = 0.40–0.90; CI = 0.64–0.77), 3.4% (8/236) with indices > 2.0 (Fig. 3A). The effect size (*n*^2^_H_) was 0.02.

The L group has exhibit the highest positivity rate at IgA ELISA positive serology [100% (8/8); median = 2.4; IQR = 1.8–3.3; CI = 1.85–3.27]; with 87.5% (7/8) of the patients showing positivity rate indices higher than 1.5 and 62.5% (5/8) showing indices > 2.0; with statistically significant differences as compared to IgM (*P* = 0.0011; Z = 2.42), IgG (*P* = 0.0002; Z = 3.44) and APGL-I (*P* = 0.0002; Z = 4.05). The serology of IgM ELISA showed positivity rate at 37.5% (3/8; median = 0.89; IQR = 0.55–1.18; CI = 0.55–1.40), with statistically significant differences as compared to APGL-I (*P* = 0.0462; Z = 1.62). The APGL-I serological assay was negative in all diagnosed patients (median = 0.45; IQR = 0.30–0.70; CI = 0.27–0.74). Anti-Mce1A IgG was positive in 25% of samples (2/8; median = 0.66; IQR = 0.31–0.97; CI = 0.36–1.01) (Fig. 3B). The small sample size (*n* = 8) relative to the number of groups (*k* = 4) should be considered when interpreting effect-size estimates.

## Discussion

The Brazilian prison system faces severe overcrowding, with an occupancy rate of 144% [13]. In the state of Alagoas, the system is designed to hold 4,631 individuals but currently houses 12,049. The Baldomero Cavalcante Male Penitentiary, where this study was conducted, was also operating beyond its designated capacity during the study period [27]. It is well recognized that leprosy is linked to inadequate sanitary and socioeconomic conditions, lower educational attainment, and environments of high overcrowding [23].

The LSQ positivity rate in our study was 28.5%, with an overall mean of 3.05 positive responses and 2.6 among patients diagnosed with leprosy. In a study conducted at a Penitentiary Progression Center in southeastern Brazil with individuals under a semi-open regime, the mean number of positive responses was lower, with 2.7 overall and 1.5 among those diagnosed with leprosy. The LSQ was positive in 75% of detected leprosy cases; however, only 34.1% were positive among individuals evaluated without a diagnosis of leprosy [14]. While the signs and symptoms described in the questionnaire may have low specificity when considered in isolation, the combination of reported items, along with the application of machine learning tools, improves accuracy and overall performance [37, 38]. Despite only limited protection, participants presented with low vaccine coverage, with 37.5% lacking a BCG scar. In previous studies conducted at other penitentiary facilities, higher rates of BCG scarring were observed [14, 24].

Comparisons between inmates in closed and semi-open prison regimes should be interpreted with caution, as differences in endemicity, social exposure, and lifestyle characteristics may influence the epidemiological and clinical profiles observed between these populations. As described by Andrews, Liu, and Croda (2023), infectious diseases continue to disproportionately affect persons deprived of liberty, as prison settings remain characterized by overcrowding, poor ventilation, limited healthcare access, and social vulnerability, conditions that favor pathogen transmission and may amplify disease spread both within prisons and in surrounding communities [39].

The NCDR in this study was 3.08%, lower than the rates reported in the female penitentiary in southeastern Brazil (3.5%) [24] and higher than that of the male penitentiary (2.7%) [14]. In 2022, the detection rate in the state of Alagoas was 8.92 per 100,000 inhabitants, and 66.6% of new cases were classified as MB [1], indicating medium endemicity [26], which may indicate underdiagnosis. When considering only patients with positive questionnaire responses, the NCDR increases to 6.52%, demonstrating the effectiveness of the LSQ in screening new leprosy cases, including within the incarcerated population, as shown in previous studies [14, 24].

The most frequently reported symptoms among patients diagnosed with leprosy were numbness in the hands and feet and nerve pain, highlighting the prominence of neurological symptoms over cutaneous changes [37, 40]. This reinforces leprosy as primarily a neural disease, especially in subtypes such as pure neural leprosy and hypochromic dimorphic forms, where clinical manifestations are centered on peripheral nerve involvement without evident skin lesions [29, 40]. Furthermore, in this form of disease presentation, asymmetrical and progressive nerve thickening is common, which complicates the differentiation of these neuropathies from other causes of peripheral neuropathy [7].

Diagnosed MB cases exhibited some GPD at diagnosis and neural involvement. These findings highlight the challenges of detection through conventional clinical screening [29, 36, 41]. In a study conducted by Silva et al. (2021), 91.2% of patients were MB and had some GPD at diagnosis, further evidencing late diagnosis within the incarcerated population [24]. This delay could go unnoticed by healthcare providers who are not specialists in dermatology or leprosy management.

The median confinement time was 40 months. Given the incubation period of leprosy, estimated between 3 and 5 years, some inmates may have acquired the disease within the prison system, while others were likely already infected before incarceration. All diagnosed cases originated from municipalities in the state of Alagoas. Most leprosy patients were aged 30–59 years or older than 60, a pattern consistent with the national trend reported in the Leprosy Epidemiological Report (2024), which indicated that 53.9% of new cases occurred in individuals aged 30–59 years and 24.6% in those older than 60 years [1].

APGL-I serology was positive in 18.6% of individuals without a leprosy diagnosis, while none of the confirmed cases tested positive. The performance of PGL-I and fusion protein LID-1 serologies in detecting leprosy varies significantly by geographic region and clinical presentation. In Northern Brazil, studies report a moderate sensitivity of 55%, which improves to 88% when serology is combined with molecular diagnostics, reflecting the complex epidemiology in hyperendemic areas [42]. Established research shows that PGL-I sensitivity is highest among MB patients and lower among PB cases, limiting its utility as an individual diagnostic tools [43, 44]. A study conducted in a highly endemic area in southeastern Brazil reported that seropositivity was associated with an 8.6-fold higher risk of leprosy among contacts and a 4.4-fold higher risk among non-contacts, despite the low test sensitivity [45].

The cases included in this study presented a low bacillary load (75% borderline and 25% pure neural), which may explain the reduced seropositivity observed for anti-PGL-I IgM antibodies. Consistent with our findings, Lima et al. (2022) reported an APGL-I seropositivity of 45.1% in a cohort predominantly composed of borderline (69%) and pure neural (5.6%) cases [46]. Also, a cohort of pure neural patients reported by Santos et al. (2017) showed 47.1% APGL-I positives [47]. In our study, the small sample size of the patient group was a limiting factor.

Serological methods with PGL-I antigen have limitations in detecting macular, neural, and early forms of the disease [45, 46]; however, the high seropositivity among individuals without clinical diagnosis highlights the need for periodic reassessment to enable early detection of new cases and indicates a marker of environmental exposure to the bacillus [8, 41, 46]. In line with these findings, Van Hooij et al. (2025) [48] demonstrated that patients with BT/TT clinical forms exhibit IgM APGL-I antibody levels comparable to healthy controls, whereas elevated levels are observed exclusively in BL/LL patients. Moreover, the use of IgG antibodies for detecting the immunodominant trisaccharide of the *M. leprae* PGL-I antigen conjugated to human serum albumin (HSA) presented limited and ineffective seropositivity, regardless of clinical form [49].

The anti-Mce1A biomarkers showed variable positivity in this population as compared to previous studies. This fact reflects the clinical profile of cases with discreet signs and symptoms and a predominance of neurological alterations. Lima et al. (2023) found higher levels of anti-Mce1A antibodies across all tested isotypes in leprosy patients and contacts from southeastern Brazil compared to healthy endemic controls. In leprosy patients, IgA anti-Mce1A ELISA positivity reached 77.5%, IgM 76.5%, and IgG 61.5%, while APGL-I serology showed only 28.0% positivity [36]. Notably, in our study, patients with pure neural forms showed positive antibodies against Mce1A for all isotypes tested, yet did not have positive APGL-I indices, even suggesting that anti-Mce1A antibodies may have greater utility for detecting early-stage and low-bacillary-burden disease. However, the small number of diagnosed cases and clinically evaluated inmates precluded a reliable assessment of diagnostic accuracy.

Anti-Mce1A seropositivity among clinically unaffected individuals reinforces the need for clinical follow-up to determine whether it predicts disease development or serves as a marker of contact with the bacillus [21, 36, 49]. In Lima et al. (2023), the general healthy population did not show anti-Mce1A serology indices greater than 2.0, suggesting that the high rates of positivity (greater than twice the cut-off) observed in non-diagnosed individuals in this study may be influenced by the region’s high endemicity and/or subclinical infection that needs to be further investigated in the long term [36, 50].

In the population evaluated in this study, we could perceive higher levels of anti-Mce1A IgA antibodies among all diagnosed patients, with more than half of those evaluated demonstrating serological indices greater than 2.0. This could indicate a subclinical disease or infection that has not yet allowed for a clinical diagnosis, suggesting that this seropositivity may be linked to a risk classification for developing the disease. Data already consolidated in two regions with different endemicity profiles in northeastern Brazil by Lima et al. (2022) and in southeastern Brazil by Lima et al. (2023) demonstrated that no healthy endemic control group presented ELISA indices for all tested antibodies greater than 2.0, and also that household contacts of patients already have antibody indices similar to those of diagnosed patients, with results ≥ 2.0 [36, 50].

The homology of the Mce1A protein with other mycobacteria requires correlation with patients’ dermatological and neurological findings. This cross-reactivity concern is reinforced when considering active TB, which is prevalent in the prison environment, with 125,105 incarcerated individuals developing TB worldwide, representing 1.0% of the global incidence, as described by Nyasulu et al. (2025) [51]. However, Lima et al. (2017) demonstrated that leprosy patients with latent tuberculosis infection (LTBI) did not show significant differences in anti-Mce1A antibody levels compared with LTBI-negative patients [21]. The high occurrence of active TB in prisons demonstrates the importance of active screening in these populations. Furthermore, none of the individuals included in this study had a diagnosis of active TB. Therefore, combining the *M. leprae* species-specific PGL-I (NDO-BSA) antigen with anti-Mce1A serology enhances the specificity of seroepidemiological screening for leprosy [50].

The clinical evaluation was performed on 260 inmates due to a riot threat at the prison, which led the team to suspend clinical exams for the remaining population to ensure participant safety. These individuals were selected as they were brought in for examination, including both LSQ-positive and LSQ-negative. Consequently, the findings may reflect underdiagnosis and underreporting of leprosy cases within the studied population. This situation aligns with known challenges of diagnosing and reporting leprosy in prison populations, where environmental and social conditions facilitate transmission, and operational constraints can limit comprehensive screening. Thus, the detected cases may represent only a fraction of the true disease burden in this setting.

A limitation of this study is the potential for selection bias, as only 260 of the 842 inmates underwent clinical evaluation, potentially affecting prevalence estimates and the observed diagnostic performance. Despite this, an active case-finding strategy was performed among both LSQ-positive and LSQ-negative individuals without prior laboratory suspicion. Nevertheless, it is worth noting that these patients would not have been diagnosed under the penitentiary’s standard clinical routine. Therefore, early and timely diagnosis was made possible exclusively by this study, enabling the identification of non-classical forms (macular forms and/or predominantly neural signs) of the disease that typically pose a challenge in clinical practice.

## Conclusion

The study confirmed the effectiveness of the LSQ as a screening tool for leprosy, consistent with previous research; however, there was no significant agreement between the LSQ and serological markers. The APGL-I test showed limited applicability due to its low positivity in detecting macular, neural, and early forms of leprosy. Nevertheless, the positivity observed among individuals without a leprosy diagnosis highlights the need for enhanced active case finding and follow-up evaluations of these inmates. The anti-Mce1A ELISA demonstrated superior positivity in detecting leprosy cases, and seropositivity in undiagnosed individuals suggests monitoring those with indices above 2.0 (indicating higher risk) and supports its potential utility as a marker of exposure to *M. leprae* in prison settings or the general population. This study contributes to the limited body of research on incarcerated populations, offering strategies to determine leprosy incidence and support new case diagnoses, aligning with the WHO agenda for disease control and the Sustainable Development Goals (SDGs), particularly SDG 3 (health and well-being).

## Supplementary Information

Below is the link to the electronic supplementary material.

Supplementary Material 1

## Data Availability

All data generated or analyzed during this study are included in this published article [and its supplementary information files].

## Abbreviations

APGL-I: Anti–Phenolic Glycolipid–I
BB: Borderline Borderline
BCG: Bacillus Calmette–Guérin
BL: Borderline Lepromatous
BT: Borderline Tuberculoid
ELISA: Enzyme–linked Immunosorbent Assay
G1D: Grade 1 Disability
G2D: Grade 2 Disability
GPD: Grade of Physical Disability
HSA: Human Serum Albumin
CI: Confidence interval
IgA: Immunoglobulin A
IgM: Immunoglobulin M
IgG: Immunoglobulin G
L: Leprosy
LL: Lepromatous
LSQ: Leprosy Suspicion Questionnaire
LTBI: Latent Tuberculosis Infection
MB: Multibacillary
Mce1A: Mammalian Cell Entry Protein 1 A
MDT: Multidrug Therapy
N: Pure Neural
NCDR: New Case Detection Rate
ND: O–BSA–Natural–octyl Disaccharide Conjugated to Bovine Serum Albumin
NL: Non Leprosy
O.D.: Optical Density
PB: Paucibacillary
PGL: I–Phenolic Glycolipid–I
RR: Relative risk
SDGs: Sustainable Development Goals
SDG 3: Sustainable Development Goal 3
SWM: Semmes–Weinstein monofilaments
TB: Tuberculosis
TT: Tuberculoid
WHO: World Health Organization
